# The value of autopsy to determine the cause of maternal deaths in Turkey

**DOI:** 10.4274/jtgga.2018.0082

**Published:** 2018-11-15

**Authors:** Hüseyin Levent Keskin, Yaprak Engin Üstün, Sema Sanisoğlu, Selma Karaahmetoğlu, Ayşe Özcan, Şevki Çelen, Saniye Tontuş, Yusuf Üstün, Veli Ongun, İrfan Şencan

**Affiliations:** 1 General Directorate of Mother and Child Health and Family Planning, Ministry of Health of Turkey, Ankara, Turkey

**Keywords:** Maternal mortality, autopsy, maternal death

## Abstract

**Objective:**

To analyze the value of autopsy reports for determining the cause of maternal deaths in Turkey.

**Material and Methods:**

In this descriptive retrospective study, the case files of 992 maternal deaths, except for accidental causes, that occurred in Turkey between 2012 and 2016 were reviewed. An autopsy examination was performed in 177 (17.8%) of the cohort. When the files were reviewed, maternal descriptive data and the cause of maternal mortality according to the autopsy reports were recorded.

**Results:**

The mean age at death was 31.5±6.6 years. No exact cause of maternal death was identified after autopsy in 44 (24.9%) of the 177 cases. An exact cause of death could be determined in 133 (75.1%); 34.5% (n=61) were due to direct causes, and 40.7% (n=72) were due to indirect causes. The leading direct causes of maternal deaths were obstetric hemorrhage (13.0%) and obstetric (pulmonary and amniotic fluid) embolism (12.4%). The main cause among the indirect causes was ruptured aortic aneurysm and/or dissection of aorta (8.5%). Among the subjects with no clinical diagnosis based on the clinical course before death (n=96), the exact cause of death could not be determined at autopsy in 19 (19.8%) cases. The exact or possible cause of death was identified at autopsy in 80.3% (n=77) cases with no clinical diagnosis. Among the cases who had antemortem diagnoses based on the clinical course (n=81), the final diagnosis at autopsy was compatible with the clinical diagnosis in 48 (59.3%) subjects.

**Conclusion:**

Maternal autopsy examination provides an exact cause of death in most cases and is still a valuable tool for understanding the cause of maternal mortality.

## Introduction

Maternal mortality is an important public health problem with socioeconomic and clinical components.

The annual number of maternal deaths decreased by 43% from approximately 532,000 in 1990 to an estimated 303,000 in 2015 (1). By 2030, every country should reduce its maternal mortality ratio (MMR) by at least two thirds from the 2010 baseline, and no country should have an MMR higher than 140 deaths per 100,000 live births (2). The MMR of Turkey between 2007 and 2009 was 19.7 per 100,000 live births (3).

The major complications that account for nearly 75% of all maternal deaths are severe hemorrhage, infections, hypertensive disorders of pregnancy, complications from delivery, and unsafe abortion. However, many maternal deaths are still not identified (2). Accurate determination of causes of maternal deaths is critical for effective prevention. Autopsy remains the gold standard evaluation for maternal deaths.

Our aim was to evaluate maternal death autopsies in a five-year period in Turkey.

## Material and Methods

In this descriptive retrospective study, the case files of all pregnancy-associated deaths recorded in Turkey between 2012 and 2016 were reviewed. Maternal deaths with autopsy results were included. Exclusion criteria were late maternal deaths and death by suicide.

The Turkish Statistical Institute (TURKSTAT) has been collecting data on the number of deaths and causes of death using the vital registration (VR) system since 2009 in details of ICD-10 codes, and underlying cause is the main concern as the World Health Organization suggests. The VR system of TURKSTAT collects data through forms that include a check box to mark whether the death was a maternal death. Maternal deaths are also discussed by the Ministry of Health. The Maternal Mortality Review Committee was formed by the Ministry of Health in 2007. All maternal deaths in Turkey must be reported to the Committee at the Ministry of Health. Identifying the cause and preventability of maternal mortality includes medical hospital records, death certificates, autopsy reports, local and national registries, and verbal autopsy. Verbal autopsy was performed routinely for every death.

The definition of maternal death was based on that used in the ICD-MM. The causes of maternal mortality are grouped into direct obstetric and indirect causes. According to the classification, maternal deaths are: Direct obstetric deaths resulting from natural obstetric complications of pregnancy, labor and puerperium or from obstetric interventions. Indirect obstetric deaths resulting from previously existing diseases or diseases that developed during pregnancy but not due to obstetric causes and worsened by pregnancy. The MMR was calculated as the number of maternal deaths to the number of births in the past one year. Clinical and pathologic autopsy results were evaluated. External examination, in situ examination, gross and microscopic examinations were performed in each case.

Maternal age, gravida, place of death (home, hospital) were recorded. The distribution of sociodemographic and clinical parameters was summarized using descriptive statistics, such as frequencies and rates, across the pregnancy continuum.

## Results

From 2012 to 2016, a total of 992 maternal deaths were recorded in Turkey. The MMR during the 5-year study period was 15.1/100,000 live births. Of these women, 177 (17.8%) underwent an autopsy.

Mean age was 31.5±6.6 (range, 16-48) years. The median gravida was 3 (range, 1-12), and the median parity was 2 (range, 1-11). In 41 cases (23.2%), the index pregnancy was the first pregnancy and 26.6% (n=47) of deaths occurred in nulliparous women. 

Death occurred while the pregnancy was ongoing in 64 (36.2%) cases, and after the pregnancy had ended in 86 (48.6%) cases. Twelve pregnant women were in the 1^st^ trimester, 24 in the 2^nd^, and 28 women were in the 3^rd^ trimester when death occurred. In 6 cases, death occurred after the pregnancy had ended in the 1^st^ or 2^nd^ trimester (spontaneous miscarriage or medical/legal termination). The remaining 86 (48.6%) women died after giving birth (29 through vaginal route, 57 via cesarean section). In 21 (11.9%) cases, the pregnancy was ended after performing perimortem cesarean section.

Twenty-six (14.7%) of the deaths happened at home, and 20 (11.3%) were admitted to the hospital as already exitus. Most of the deaths (n=131, 74%) were pronounced at a hospital. Sixty women were admitted under cardiopulmonary resuscitation after arrest or with general condition disturbance. In 71 cases, death occurred when they were in the hospital for giving birth or under the treatment for any disorders during the pregnancy or in the postpartum period.

The cause of maternal death was undetermined in 44 cases (24.9%) at the end of autopsy (Table 1).

Although in 44 (24.9%) out of 177 cases the exact cause of maternal death was undetermined at the end of autopsy, the exact cause of death could be detected in 133 (75.1%) (Table 1); 34.5% (n=61) were due to direct causes, and 40.7% (n=72) were due to indirect causes. The leading causes of the direct maternal death were obstetric hemorrhage (13.0%) and obstetric (pulmonary and amniotic fluid) embolism (12.4%). The main cause among the indirect causes was ruptured aortic aneurysm and/or dissection of aorta (8.5%) (Table 1).

Among the subjects who had no clinical diagnosis based on the antemortem clinical course before death (n=96), the exact cause of death could not be determined after autopsy in 19 (19.8%) cases. However, the exact or possible cause of death was identified in 80.3% (n=77) of cases (Table 2). The most common cause of death in those cases were ruptured aortic aneurysm and/or dissection of aorta (n=15) and pulmonary embolism (n=14) (Table 2).

Among the women who had an antemortem diagnosis based on the clinical course (n=81), the final diagnosis was compatible with the clinical course in 48 (59.3%) cases, and the autopsy diagnosis was incompatible with the clinical diagnosis in 8 (9.9%) cases. However, in 25 (30.9%) out of 81 cases with an antemortem diagnosis based on clinical findings, the exact cause of death could not be determined at autopsy.

In 23 (13%) cases, although the exact cause of the death was clearly defined, judicial autopsy was performed because of medicolegal issues.

Of the 7 cases diagnosed as amniotic fluid embolism (AFE) after autopsy, five were diagnosed as embolism with clinical findings before death occurred; however, no specific clinical diagnosis was considered as a differential diagnosis during the antemortem period in two cases. AFE was considered according to the antemortem clinical course in 8 cases -out of 44 with no definite cause of death were determined at autopsy- although the exact cause of death could not be identified at autopsy.

In addition, in 7 out of 44 cases with no exact cause of death determined at autopsy, epilepsy featured in their medical history and the cause of maternal mortality was accepted as sudden, unexpected death in epilepsy.

## Discussion

Every year approximately 300,000 women die because of the complications of giving birth (4). Determination of the etiologies of maternal mortality should be a priority to achieve a significant reduction in maternal mortality. A reliable ascertainment of the causes of maternal mortality requires an autopsy (5). Its value was revealed in the study of Sonderegger-Iseli et al. (6) with clinical discrepancies in up to 30% of cases.

In our study, maternal autopsy improved the understanding of the cause of deaths in nearly half of the cases.

Castillo et al. (7) found that the minimally invasive autopsy method could be an important implementation to decide the etiologies of maternal death, especially for indirect maternal mortality causes, most of which are infectious diseases. Minimally invasive autopsy, which is made up of the evaluation of samples of basic organs and fluids in terms of histology and microbiology, could improve the value of the currently used procedures including verbal autopsies and clinical records, which have been revealed to have a high level of imprecision. Hasegawa et al. (8) reported that in most cases autopsy provided an exact cause of death, the necessity of autopsies should be more widely accepted, and autopsies should be performed more frequently in Japan.

Kavatkar et al. (9) showed that certain final pathogenetic mechanisms such as disseminated intravascular coagulation, acute renal failure, shock, congestive cardiac failure and hepatic encephalopathy led to maternal death. In the present study, the most frequent cause of mortality found at autopsy were aortic aneurysm rupture and pulmonary embolism.

Autopsies of maternal death have greater importance than other deaths because these reports are used to make recommendations for ameliorating clinical obstetric practice and defining the cause of death.

In conclusion, maternal autopsy examination provides an exact cause of death in most cases and is still a valuable tool for understanding the cause of maternal mortality.

## Figures and Tables

**Table 1:**
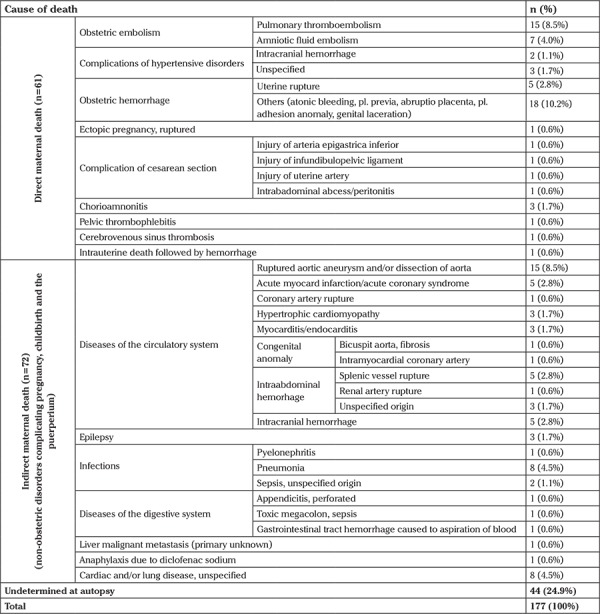
Causes of maternal deaths based on autopsy results (n=177)

**Table 2:**
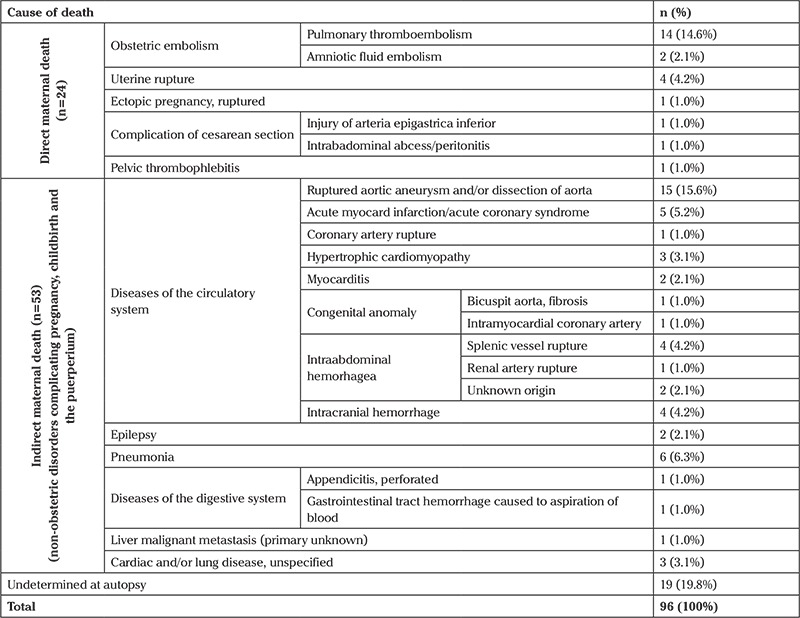
The causes of maternal deaths identified by maternal autopsy without any clinical course information or suspicion before death (n=96)
